# Host choice and multiple blood feeding behaviour of malaria vectors and other anophelines in Mwea rice scheme, Kenya

**DOI:** 10.1186/1475-2875-7-43

**Published:** 2008-02-29

**Authors:** Simon M Muriu, Ephantus J Muturi, Josephat I Shililu, Charles M Mbogo, Joseph M Mwangangi, Benjamin G Jacob, Lucy W Irungu, Richard W Mukabana, John I Githure, Robert J Novak

**Affiliations:** 1 Human Health Division, International Centre of Insect Physiology and Ecology P. O. Box 30772-00100, Kenya, Africa; 2 School of Biological Sciences, University of Nairobi, Nairobi, Kenya, Africa; 3 Department of Medicine, William C. Gorgas Center for Geographic Medicine, University of Alabama at Birmingham, Birmingham AL. 35294, USA; 4 Department of Zoology, Jomo Kenyatta University of Agriculture and Technology, Nairobi, Kenya, Africa; 5 Kenya Medical Research Institute, Centre for Geographic Medicine Research – Coast, Kenya, Africa

## Abstract

**Background:**

Studies were conducted between April 2004 and February 2006 to determine the blood-feeding pattern of *Anopheles *mosquitoes in Mwea Kenya.

**Methods:**

Samples were collected indoors by pyrethrum spay catch and outdoors by Centers for Disease Control light traps and processed for blood meal analysis by an Enzyme-linked Immunosorbent Assay.

**Results:**

A total of 3,333 blood-fed *Anopheles *mosquitoes representing four *Anopheles *species were collected and 2,796 of the samples were assayed, with *Anopheles arabiensis *comprising 76.2% (n = 2,542) followed in decreasing order by *Anopheles coustani *8.9% (n = 297), *Anopheles pharoensis *8.2% (n = 272) and *Anopheles funestus *6.7% (n = 222). All mosquito species had a high preference for bovine (range 56.3–71.4%) over human (range 1.1–23.9%) or goat (0.1–2.2%) blood meals. Some individuals from all the four species were found to contain mixed blood meals. The bovine blood index (BBI) for *An. arabiensis *was significantly higher for populations collected indoors (71.8%), than populations collected outdoors (41.3%), but the human blood index (HBI) did not differ significantly between the two populations. In contrast, BBI for indoor collected *An. funestus *(51.4%) was significantly lower than for outdoor collected populations (78.0%) and the HBI was significantly higher indoors (28.7%) than outdoors (2.4%). Anthropophily of *An. funestus *was lowest within the rice scheme, moderate in unplanned rice agro-ecosystem, and highest within the non-irrigated agro-ecosystem. Anthropophily of *An. arabiensis *was significantly higher in the non-irrigated agro-ecosystem than in the other agro-ecosystems.

**Conclusion:**

These findings suggest that rice cultivation has an effect on host choice by *Anopheles *mosquitoes. The study further indicate that zooprophylaxis may be a potential strategy for malaria control, but there is need to assess how domestic animals may influence arboviruses epidemiology before adapting the strategy.

## Background

*Anopheles *mosquitoes are important vectors of malaria and several arboviruses. Although more than 500 species of *Anopheles *have been described, only less than one third are considered vectors, and one or two species are known to be major drivers of disease transmission dynamics in a given area [1]. The rate of disease transmission is dependent on vector distribution, abundance and lifespan, degree of host-vector-pathogen contact, susceptibility of the vector to the pathogen, and the effects of the pathogen on survivorship of both the vector and the host. These factors are further dependent on local ecologic factors such as local climatic conditions, topography, water table, occurrence and diversity of larval habitats and human lifestyles [2,3].

For a mosquito to transmit an infection to humans, it must take at least two blood meals to facilitate uptake of the pathogens, and eventual transmission to a susceptible human. The degree of human-vector contact is, therefore, considered one of the most important components of disease transmission and is used in planning and evaluating the risk of vector-borne disease and the impact of vector control measures [4]. *Anopheles *mosquitoes with preference for human blood are considered important vectors of malaria and Bancroftian filariasis, and those with multiple blood meal hosts may increase the rate of arboviruses transmission [5,6]. The choice of blood meal is influenced by several factors including host availability, nutritional requirements, intrinsic host preferences of the species, and vector density [7-10].

Irrigated rice agro-ecosystems are considered important "hotspots" for mosquito-borne diseases because of the numerous mosquito species present. Worldwide, more than 89 species of *Anopheles *are associated with rice cultivation [11], and at least 23 species occur in a variety of aquatic habitats present in African rice agro-ecosystems [5,12-14]. In Africa, the risk of human exposure to disease transmission by majority of these species is not fully understood because most studies are restricted to the main vectors of malaria and Bancroftian filariasis [15-17]. Review of the scanty literature indicates that rice land *Anopheles *mosquitoes tend to be highly zoophilic. Such findings have been documented for *Anopheles gambiae s.s*., *Anopheles arabiensis*, *Anopheles funestus*, *Anopheles pharoensis *and *Anopheles rufipes *[9,18-20]. Researchers have, therefore, suggested that zooprophylaxis could be an effective strategy for controlling malaria in these areas [15,21,22]. This method has been used successfully in some parts of Africa [7] and is also considered to have played a significant role in reduction of malaria in Europe and USA earlier in the last century [23]. However, studies have shown that the pattern of host choice and preference by *Anopheles *mosquitoes is site-specific and dependent on local ecologic factors [4]. It is, therefore, conceived that decisions regarding zooprophylaxis can only be made based on local context of available hosts and blood feeding preferences [24]. The aim of the current study was to determine the blood-feeding patterns of *Anopheles *mosquitoes in Mwea Rice Irrigation Scheme in central Kenya.

## Methods

### Study area

The studies were conducted in Mwea Rice Irrigation Scheme in central Kenya, 100 km North-east of Nairobi. The study area has been described previously [25,18,15,14]. Mwea Rice Scheme covers an area approximately 13,600 ha consisting of forty villages and 150,000 people in 2,500 households. Eight study sites were selected for the study based on rice growing practices and water management. These included six villages within the rice scheme (Ciagi-ini, Mbuinjeru, Rurumi, Karima, Kiuria and Kangichiri) where 75% of each village land is under rice cultivation, and two villages outside the scheme (Kiamachiri and Murinduko). Kiamachiri is located immediately outside the scheme and 20% of the village land is under rice cultivation. Murinduko is approximately 15 km away from the scheme and only less than 5% of the village land is under rice cultivation because most of the land is hilly and unsuitable for rice cultivation. Rice cultivation in the villages within the scheme follows a definite cropping cycle as determined by the National Irrigation Board (planned rice cultivation) whereas in Kiamachiri and Murinduko rice cultivation continues throughout the year as long as water is available (unplanned rice cultivation). Because only a small portion of land in Murinduko was under rice cultivation, we considered this village to be non-irrigated. Cattle, goats and chicken are the main domestic animals kept in the study area. Majority of the houses are mud-walled with iron roofing and unscreened eaves and windows.

Eight species of *Anopheles *are known to occur in the area with *An. arabiensis *as the dominant species [14] and the only member of the *An. gambiae *complex present [26]. Previous studies have shown that both agricultural and environmental factors play a significant role in determining the occurrence and abundance of these species [27,28]. *Anopheles arabiensis *and *An. funestus *are the known vectors of malaria in the area [18,15].

### Mosquito collection

The sampling frame for adult mosquito collection extended between April 2004 and February 2006. For logistic purposes samples for Mbuinjeru, Kiamachiri and Murinduko were collected between April 2004 and March 2005 and those for Ciagi-ini, Rurumi, Karima, Kiuria and Kangichiri were collected between March 2005 and February 2006. Indoor-resting mosquitoes were collected by pyrethrum spray catch (PSC) method [29] and outdoor populations were collected by Centers for Disease Control (CDC) miniature light traps (J.W. Hock Ltd, Gainesville, FL., USA). A detailed explanation of the sampling strategy has been described elsewhere [14].

### Laboratory processing

All *Anopheles *mosquitoes collected by PSC and CDC light traps were transported to the laboratory and sorted by sex and species using morphological characteristics [30]. The females were further classified into their respective blood feeding stages (unfed, blood-fed, semi-gravid and gravid) by examining their abdomen under a dissecting microscope [29]. All blood-fed mosquitoes from each collection were preserved in labeled vials containing anhydrous calcium sulphate.

### Blood meal identification

Samples of blood fed mosquitoes were cut transversely between the thorax and the abdomen, and the posterior portions containing the blood meal were placed individually in labeled vials. The abdomen of each mosquito was ground in 50 μl of phosphate-buffered saline (PBS) with subsequent addition of 950 μl of PBS and then stored at -20°C. Blood meals were identified by a direct enzyme-linked immunosorbent assay (ELISA) using anti-host (IgG) conjugates (Kirkegaard and Perry, Gaithersburg, MD) against human, bovine and goat [31]. All blood meal samples were screened simultaneously for human, bovine and goat antibodies.

### Data analysis

Data were entered in Microsoft excel and analyzed using Epi-Info^® ^software version 3.4.1 (CDC Atlanta, Georgia USA). Chi-square and Fishers exact tests were used (as appropriate) to compare the differences in the human blood index (HBI) and bovine blood index (BBI) between indoor and outdoor collected populations of *An. arabiensis *and *An. funestus*. The HBI and BBI were calculated as the ratio of blood-fed mosquito samples that had fed on human and cattle respectively to the total tested. Chi-square test was also used to compare the differences in HBI for *An. arabiensis *and *An. funestus *among planned, unplanned, and non-irrigated agro-ecosystems. Data for the six villages within the rice scheme (planned rice) were pooled before analysis was done.

## Results

In total, 3,333 blood-fed *Anopheles *mosquitoes collected indoors and outdoors at eight sites in Mwea, Kenya were tested by ELISA for host blood meal and 2,796 (83.9%) of the samples could be identified. These comprised *An. arabiensis *(n = 2,542), *An. funestus *(n = 222), *Anopheles coustani *(297), and *An. pharoensis *(n = 272). Overall, majority of the blood meals were of bovine origin (69.9%) followed by human (8.1%). The remaining blood meals were from goat (0.4%) and mixed blood meals from bovine and goat (3.0%), human and bovine (2.0%), human and goat (0.1%) and human, bovine and goat (0.3%) (Table 1).

**Table 1:** Blood-meal sources of *Anopheles *mosquitoes in Mwea Rice Irrigation Scheme, in central Kenya.

Species	Location	# tested	Human (%)	Bovine	Goat	Human/bovi ne	Human/goat	Bovine/goat	Human/bovine/goat	Unknown
*An. arabiensis*	Indoors	2467	194 (7.9)	1771 (71.8)	3 (0.1)	37 (1.5)	0 (0)	21 (0.9)	6 (0.2)	435 (17.6)
	Outdoors	75	5 (6.7)	31 (41.3)	0 (0)	4 (5.3)	1 (1.3)	5 (6.7)	(0) 0	29 (38.7)
	Overall	2542	199 (7.8)	1802 (70.9)	3 (0.1)	41 (1.6)	1 (0.04)	26 (1.0)	6 (0.2)	464 (18.3)
*An. funestus*	Indoors	181	52 (28.7)	93 (51.4)	1(0.6)	1 (0.6)	0 (0)	13 (7.2)	0 (0)	21 (11.6)
	Outdoors	41	1 (2.4)	32 (78)	0 (0)	2 (4.9)	(0) 0	3 (7.3)	1 (2.4)	2 (4.9)
	Overall	222	53 (23.9)	125 (56.3)	1 (0.5)	3 (1.4)	0 (0.0)	16 (7.2)	1 (0.5)	23 (10.4)
*An. pharoensis*	Indoors	3	0 (0)	1 (100)	0 (0)	0 (0)	0 (0)	0 (0)	0 (0)	0 (0)
	Outdoors	269	3 (1.1)	189 (70.3)	6 (2.2)	13 (4.8)	0 (0)	31 (11.5)	2 (0.7)	25 (9.3)
	Overall	272	3 (1.1)	192 (70.6)	6 (2.2)	13 (4.8)	0 (0)	31 (11.4)	2 (0.7)	25 (9.2)
*An. coustani*	Indoors	1	0 (0)	1 (100)	0 (0)	0 (0)	0 (0)	0 (0)	0 (0)	0 (0)
	Outdoors	296	16 (5.4)	211 (71.3)	3 (1.0)	9 (3.0)	3 (1)	28 (9.5)	1 (0.3)	25 (8.4)
	Overall	297	16 (5.4)	212 (71.4)	3 (1.0)	9 (3.0)	3 (1.0)	28 (9.4)	1 (0.3)	25 (8.4)
All species combined	Indoors	2652	246 (9.3)	1868 (70.4)	4 (0.2)	38 (1.4)	0 (0)	34 (1.3)	6 (0.2)	456 (17.2)
	Outdoors	681	25 (3.7)	463 (68)	9 (1.3)	28 (4.1)	4 (0.6)	67 (9.8)	4 (0.6)	81 (11.9)
	Overall	3333	271 (8.1)	2331 (69.9)	13 (0.4)	66 (2.0)	4 (0.1)	101 (3.0)	10 (0.3)	537 (16.1)

Eighty two percent of *An. arabiensis *samples (n = 2,542) were positive for at least one of the three host blood meals tested. The majority of them had predominantly fed on cattle (70.9%), to a lesser extent on humans (7.8%) and rarely on goats (0.1%). Seventy-four samples (2.84%) proved to be of mixed origin mainly from human and bovine (1.6%) and bovine and goat (1.0%) (Table 1). Most of the blood fed *An. arabiensis *females were collected indoors as opposed to outdoors (Table 1). Although the human blood index for this species did not differ significantly between indoor and outdoor collected populations (Fisher exact test = 0.57 df = 1 *P *= 0.425), the bovine blood index was significantly higher among indoor (71.8%) than outdoor collected (41.3%) samples (χ^2 ^= 6.3 df = 1 *P *< 0.05). In contrast, the proportion of samples containing mixed blood meals was higher among outdoor than indoor collected mosquitoes (Table 1).

For *An. funestus*, 89.6% (n = 222) of the blood meal samples were successfully identified and shown to consist of at least one of the three hosts tested. The majority of blood meals were from cattle (56.3%) and humans (23.9%), and only one blood meal was of goat origin. Mixed blood meals mainly of bovine and goat (7.25%), and human and bovine (1.4%) were also obtained (Table 1). The number of blood fed *An. funestus *was four-fold higher indoors than outdoors. The human blood index for indoor collected *An. funestus *was 28.7% and significantly higher than 2.4% for outdoor populations (Fisher exact test = 9.73 df = 1 *P *< 0.05). In contrast, bovine blood index of 51.4% for indoor collected populations was significantly lower than 78.0% for outdoor collected populations (χ^2 ^= 13.78, df = 1 *P *< 0.05). Mixed blood meals were common in both indoor and outdoor samples.

With a single exception, all blood-fed *An. coustani *were collected outdoors. This species had predominantly fed on cattle (71.4%), over humans (5.4%) or goats (1.0%). Mixed blood meals mainly of bovine and goat (9.5%), human and bovine (3.0%), and human and goat (1.0%) were also obtained. The single specimen from indoor collections had feed on cattle and 25 blood meal samples were of unknown sources.

*Anopheles pharoensis *also fed predominantly on cattle (70.3%) over human (1.1%) or goat (2.2%). Mixed blood meals were mainly of bovine and goat (11.4%), and human and bovine (4.8%) origin. All but three samples of *An. pharoensis *were collected outdoors. The three samples collected indoors had fed on cattle.

When the blood feeding patterns of *An. arabiensis *and *An. funestus *were separated by village, the human blood index (HBI) for *An. funestus *was significantly higher in the villages outside the rice scheme (Kiamachiri and Murinduko) than those within the scheme (χ^2 ^= 35.02 df = 1 P < 0.05). HBI for this species (*An. funestus*) was also significantly higher in Murinduko than in Kiamachiri (χ^2 ^= 11.27 df = 1 P < 0.05). The HBI for *An. arabiensis *was significantly higher in Murinduko compared with the other villages (Table 2) (χ^2 ^= 25.86, df = 1, *P *< 0.001).

**Table 2:** Proportion of human and bovine blood meals for *Anopheles arabiensis *and *An. funestus *collected at eight sites representing three agro-ecosystems in Mwea, Kenya.

		*An. arabiensis*			*An. funestus*		
Agro-ecosystem	Village	No. tested*	% Human	% Bovine	No. tested	% Human	% Bovine

Planned rice	Ciagi-ini	227	2.2	84.1	30	0.0	96.7
	Kangichiri	240	2.1	51.7	7	14.3	71.4
	Karima	265	7.5	76.6	26	11.5	88.5
	Kiuria	309	6.1	78.0	48	0.0	93.8
	Rurumi	214	7.9	80.4	16	6.3	93.8
	Mbui-njeru	815	9.3	78.2	19	10.5	73.7
	Overall	2070	6.9	75.7	146	4.8	89.7
Unplanned rice	Kiamaciri	338	11.5	73.7	11	27.3	63.6
Non-irrigated	Murinduko	134	49.3	43.3	65	72.3	9.2

* Results for indoor and outdoor collected mosquitoes were pooled together

## Discussion

Out of the 8 species of *Anopheles *known to occur in the Mwea Rice Irrigation Scheme [18,15], four of them were examined for blood meal sources and found to have higher preference for bovine over human hosts. Similar results have been reported in different rice growing areas in the African continent [18-20,15]. Rice cultivating areas are often associated with higher mosquito densities and human communities in these areas enforce the use of bed nets and other protective measures against biting mosquitoes [32]. Consequently, mosquitoes revert to feeding on domestic animals because humans are not easily accessible. These results demonstrated that majority of *An. arabiensis *gained entry into the house after feeding outdoors on bovine and that indoor-collected mosquitoes had no advantage over outdoor collected populations in terms of access to human blood meals. These findings confirmed that protection against mosquito bites is indeed one of the factors accounting for zoophilic tendency of *Anopheles *mosquitoes in rice irrigated areas. Interestingly, 16% of the blood meal samples were not from any of the three hosts tested an indication that anophelines in this area have a wide host range. These findings highlight the need to include a variety of possible hosts when conducting mosquito host choice studies.

Reduced anthropophily of *Anopheles *mosquitoes in rice cultivating areas has been suggested as one of the factors responsible for the low levels of malaria transmission in these areas despite the presence of higher vector densities [15,18-20,32]. Zooprophylaxis is, therefore, considered a potential malaria control strategy in rice growing areas of Africa [15]. However, it should be noted that *An. funestus *differed from *An. arabiensis *in a way that is likely to affect malaria epidemiology and the impact of zooprophylaxis on malaria control. *Anopheles funestus *was substantially more anthropophilic and endophagic than *An. arabiensis*. These observations indicate that inhabitants in the study area are at a greater risk of exposure to malaria transmission by *An. funestus *than by *An. arabiensis *and that *An. funestus *may not be a good candidate of zooprophylaxis. These findings therefore, emphasize the idea of Hadis *et al *[2] that conclusions regarding zooprophylaxis cannot be generalized for all mosquito species. *Anopheles funestus s.l*. consists of nine species that are difficult to distinguish by morphological characteristics [33]. All except *An. funestus s.s *and to some extent *An. rivulorum *are believed to be zoophilic and non-vectors [34]. Due to limitation in resources, the species composition within the *An. funestus *group was not determined in the current study. Previous studies [35,36] using indoor collected samples found the species composition of the *An. funestus *complex within the Mwea Rice Scheme to be comprised mainly of *An. parensis *(99%) and *An. leesoni *(<1%). Based on these studies and the current HBI results it is likely that *An. parensis *was the dominant species in villages within the irrigation scheme as opposed to *An. funestus s.s*. in the non- irrigated areas. However, further studies are necessary to define the species composition of the *An. funestus *complex in the area and their role in malaria transmission. Such studies should take into account the indoor and outdoor resting localities of members of this species complex.

A considerable proportion of all *Anopheles *species examined contacted more than one host during a single gonotrophic cycle. This is common among mosquitoes and its epidemiological significance is controversial [2,37,38]. In malaria transmission, the loss of certain number of sporozoites in non-human hosts during mixed feeding could be of importance in malaria control. In fact, domestic animals have been associated with a decrease in malaria transmission because of zoophilic deviation [37]. For this reason, some African communities intentionally keep cattle near or inside houses to divert mosquitoes from humans to cattle [38]. On the other hand, the presence of domestic animals may enhance or suppress transmission of arboviruses depending on whether the vector feeds on potential or unimportant hosts. In Australia, close proximity of humans to domestic pigs and high mosquito densities was associated with an outbreak of Japanese encephalitis (JE) [39]. In contrast, multiple feeding from dead end hosts (cattle) was associated with a decrease in prevalence of JE in India [40]. Based on these findings, it is possible that multiple feeding observed in this study could reduce malaria transmission but potentially enhance transmission of arboviruses such as West Nile Virus and Rift Valley Fever Virus which is emerging as an important public health problem in Kenya and other African countries [41-43]. In the past, several *Anopheles *species including *An. pharoensis *and *An. coustani *have been associated with transmission of the two arboviruses [44,45]. Members of the *An. gambiae s.l*. and *An. funestus s.l *are also known to transmit O'nyong-nyong virus in east Africa [46]. These observations coupled with high vector densities in the study area suggest that arboviral epidemics could easily be initiated even by arrival of a single viremic individual [47]. Further studies are recommended to evaluate the potential role of domestic animals in arboviruses transmission in similar areas.

The results of this study also demonstrated that land use had significant impact on blood feeding pattern of *An. arabiensis *and *An. funestus*. Anthropophily for *An. funestus *was lowest within the rice scheme, moderate in the unplanned rice agro-ecosystem, and highest in the non-irrigated agro-ecosystem and that of *An. arabiensis *was significantly higher in the non-irrigated agro-ecosystem than in the other agro-ecosystems. The numbers of *Anopheles funestus s.l*. were low in all agro-ecosystems especially when compared to those of *An. arabiensis*. For unknown reasons, probably related to larval habitat characteristics, *An. funestus *is a rare species in African rice agro-ecosystems except in Ahero Rice Scheme, Kenya and plateaus of Madagascar [48-50]. Previous studies in the study area revealed that mosquito densities increased proportionally with increasing area under rice cultivation [14]. The area under rice cultivation in the villages within the rice scheme was approximately four and 15 times greater than in Kiamachiri and Murinduko, respectively. It is, therefore, logical to expect increasing anthropophilic tendency of these mosquito species as you move away from rice the scheme because protection against mosquito bites is directly proportional to mosquito densities [32,19,15,20]. It is, however, worth to note that the limited resources neither allowed for replication of villages outside the rice scheme nor collection of mosquito samples at the same time frame in all villages. The villages within the irrigation scheme did not show great disparity in mosquito feeding behavior and was consequently assumed that those outside the irrigation scheme would have similar characteristics and effect on mosquito host choice. Hence, the single villages outside the scheme would present the unique features associated with land use patterns in relation to malaria vectors and their choice of blood meal source. Nonetheless, further studies conducted simultaneously in equal number of villages for each agro-ecosystem and at the same sampling period would be more informative.

The current study adopted pyrethrum spray catch (PSC) and CDC light trap methods for collection of indoor and outdoor resting mosquito populations, respectively. This was necessitated by the need to capture large representative samples of all *Anopheles *species present in the study area for evaluation of the feeding behaviour and host choices in different environments. The strongly exophagic and exophilic mosquito species such as *An. pharoensis *[51] would be captured by light traps at night when most such species are active. On the other hand, some members of similar or different species spend most or part of their time resting indoors like *An. funestus s.l*. and thus majority would be captured entirely or partly through PSC method. The combination of the two mosquito collection methods with individual bias towards different mosquito species helped increase the diversity and density of the species collected and tested for blood feeding behaviour.

## Conclusion

This study has demonstrated that *Anopheles *mosquitoes in the Mwea Rice Scheme are highly zoophilic and that multiple feeding within the same gonotrophic cycle is common among these species. The study has further revealed that the degree of anthropophily by malaria vectors is directly proportion to the area of land under rice cultivation. By pooling together available literature and the findings of this study, it can be inferred that zooprophylaxis is a potential malaria control strategy in Mwea and similar areas, but it may also enhance arbovirus transmission. It is, therefore, essential to evaluate the impact of zooprophylaxis on arboviruses transmission before adopting it as a malaria control strategy.

## Competing interests

The author(s) declare that they have no competing interests.

## Authors' contributions

SMM conducted the field studies, analyzed the data and wrote the manuscript. EJM participated in development of the study, interpretation of the data and provided a critic review of the manuscript. JIS and CMM provided scientific guidance in data collection, analysis and manuscript preparation and planning, and implementation of day-to-day field and laboratory activities. JMM, BJ, LWI and RWM, offered scientific guidance in data analysis and manuscript preparation. JG and RN provided overall supervision of the study and preparation of manuscript. All authors actively contributed to the interpretation of the findings and development of the final manuscript and approved the final manuscript.
